# Comparative evaluation of Ordered Subset Expectation Maximization and Bayesian Penalized Likelihood algorithms for PET/CT image reconstruction in various malignancies using ^18^F-FDG and ^68^Ga-PSMA-11 tracers

**DOI:** 10.3389/fonc.2025.1597919

**Published:** 2025-08-29

**Authors:** Arvin Naeimi, Sara Harsini, Ramin Akbarian Aghdam, Ran Klein, Farzad Abbaspour

**Affiliations:** ^1^ Department of Radiology, McGill University Health Center, McGill University, Montreal, QC, Canada; ^2^ Student Research Committee, School of Medicine, Guilan University of Medical Sciences, Rasht, Iran; ^3^ Department of Medical Imaging, University of Toronto, Toronto, ON, Canada; ^4^ Division of Nuclear Medicine, Department of Medicine, The Ottawa Hospital and University of Ottawa, Ottawa, ON, Canada; ^5^ Division of Nuclear Medicine, Department of Radiology, McGill University Health Center, McGill University, Montreal, QC, Canada

**Keywords:** Bayesian Penalized Likelihood, BPL, Ordered Subset Expectation Maximization, OSEM, image reconstruction, Positron Emission Tomography-Computed Tomography, PET/CT, standardized uptake value

## Abstract

**Purpose:**

This study compares the Ordered Subset Expectation Maximization (OSEM) and Bayesian Penalized Likelihood (BPL) algorithms for Positron Emission Tomography/Computed Tomography (PET/CT) image reconstruction using ^18^F- fluorodeoxyglucose (FDG) and ^68^Ga-labeled Prostate-Specific Membrane Antigen(^68^Ga-PSMA-11) tracers.

**Methods:**

A retrospective analysis was conducted on 33 patients with various malignancies, including 25 undergoing ^18^F-FDG PET/CT scans and 8 undergoing ^68^Ga-PSMA-11 PET/CT scans. Scans were reconstructed using both OSEM and BPL (Q.Clear) algorithms, evaluating key metrics such as Standardized Uptake Value (SUV)max, SUVpeak, background SUV, and tumor-to-background ratio (TBR).

**Results:**

Thirty-three patients (mean age: 67.53 ± 11.78 years) with 100 lesions (80 FDG, 20 PSMA) were analyzed. In the FDG group, significant differences were observed in lesion SUVpeak, liver SUVpeak, SD of liver SUVmean, bladder SUVmean, SD of bladder SUVmean, and TBR, with BPL generally producing higher values except for liver SUVpeak, SD of liver SUVmean, and SD of bladder SUVmean. In the PSMA group, BPL enhanced most metrics except for the SD of liver SUVmean and the SD of bladder SUVmean. While strong linear correlations between BPL and OSEM metrics were observed (Pearson r>0.85 for most parameters), Bland-Altman analysis revealed wide limits of agreement, particularly for TBR in the PSMA group (-13.00 to 23.89), indicating substantial variability in absolute values between methods. Assessment of the relationship between lesion volume and SUVmax differences (BPL–OSEM) revealed a weak, non-significant negative correlation in the total cohort(r = – 0.14, p = 0.16) and in the FDG subgroup(r= – 0.14, p = 0.24).

**Conclusion:**

Both reconstruction methods demonstrate clinical utility, with BPL producing statistically higher values for several quantitative metrics, such as SUVmax and TBR, without markedly improving lesion detectability. While strong correlations were observed between BPL and OSEM values, the wide limits of agreement, particularly for TBR in PSMA imaging, suggest these methods may not be directly interchangeable in longitudinal studies. Harmonization strategies may help reduce inter-method variability and improve scan comparability in longitudinal or multicenter settings. Prospective approaches, such as reconstruction-specific reference ranges or scaling factors, could further support harmonization efforts in clinical trials. For longitudinal monitoring, consistent use of the same reconstruction method is recommended to ensure reliable quantification.

## 1 Introduction

Positron Emission Tomography (PET), combined with Computed Tomography (CT), is extensively utilized in the initial diagnosis, staging, therapeutic response evaluation, and prognostication of numerous malignant diseases. The quantitative aspect of PET, particularly the standardized uptake values (SUVs), plays a crucial role in the early prediction of tumor response and patient outcomes, thereby guiding clinical decision-making (1). However, several factors, including physical, biological, and technical, can affect the accuracy of quantification in PET imaging (2). Therefore, standardization of parameters influencing quantification is essential before their application as biomarkers. Standardization is especially essential for comparison of biomarker data, whether longitudinally on the same PET/CT device or between PET/CT devices.

One of the critical factors influencing the accuracy of quantitative parameters is the image reconstruction method. The Ordered Subsets Expectation Maximization (OSEM) algorithm has been the predominant method for PET image reconstruction. OSEM is an iterative statistical algorithm that requires the definition of subsets and iterations for reconstructing images (3). Although increasing the number of subsets and iterations can theoretically improve image accuracy, it also amplifies noise, which can degrade image quality and lead to inaccuracies in quantification and segmentation. The introduction of time-of-flight (TOF)-based systems has partially mitigated these issues by achieving total activity convergence at lower iterations (4, 5).

To address these limitations, the Block Sequential Regularized Expectation Maximization (BSREM) algorithm, also known as Bayesian penalized likelihood (BPL) by General Electric (GE Healthcare, Milwaukee, WI, USA), has been introduced. BSREM is a BPL method that incorporates a noise suppression mechanism through a penalty term weighted by a parameter β (6). This approach enables fully convergent iterative reconstruction, resulting in enhanced image contrast and noise suppression compared to OSEM (7). The core advantage of this activity-dependent noise control function, known as the relative difference penalty (6), is its ability to de-noise the image while preserving edges, allowing BSREM to achieve (practical) convergence. Additionally, BPL incorporates a point-spread function (PSF) model that further improves spatial resolution (6, 8, 9).

Despite the theoretical advantages of BSREM, its clinical application has been limited by computational demands and accessibility issues. Recent advancements, however, have facilitated its integration into clinical practice, with initial studies indicating either comparable or superior overall image quality, contrast recovery, and lesion detectability compared to OSEM (10–17). However, differences in image processing techniques can lead to inconsistent measurements of common PET biomarkers across various locations or equipment. Detailed comparisons of these two reconstruction algorithms in clinical practice are still limited.

This retrospective analysis aims to compare the OSEM and BPL (Q.Clear) reconstruction algorithms concerning their impact on quantitative parameters in patients undergoing either ^68^Ga-labeled Prostate-Specific Membrane Antigen (^68^Ga-PSMA-11) or ^18^F-Fluorodeoxyglucose (^18^F-FDG) PET/CT scans.

## 2 Materials and methods

### 2.1 Patients

This study retrospectively analyzed a cohort of 33 consecutive patients with various malignancies who underwent either ^18^F-FDG PET/CT or ^68^Ga-PSMA-11 PET/CT at the Ottawa Hospital between October 28, 2022, and March 27, 2023. Only patients for whom both OSEM and BPL reconstructions were available were included in the study. All procedures complied with national regulations and the principles of the 1964 Helsinki Declaration and its subsequent amendments. The Ottawa Hospital Institutional Review Board approved this study and waived the requirement for informed consent due to its retrospective nature.

### 2.2 Imaging protocol

Patients underwent scanning as part of their routine clinical evaluations using a hybrid PET/CT system (Discovery 710, GE Healthcare, Milwaukee, USA). For ^18^F-FDG PET/CT scans, patients fasted for a minimum of 6 hours prior to image acquisition, maintaining venous blood glucose levels below 9.0 mmol/L. Each patient received an intravenous injection of 4.99 MBq/kg ^18^F-FDG, with PET emission images captured 60 minutes post-injection. For ^68^Ga-PSMA-11 PET/CT scans, PET emission images were obtained 60 minutes after administering a bodyweight-adjusted dose of 2–4 MBq/kg ^68^Ga-PSMA-11. Patients were instructed to void immediately before the ^68^Ga-PSMA-11 study.

For both ^18^F-FDG PET/CT and ^68^Ga-PSMA-11 studies, a scout view and a non-contrast-enhanced low-dose spiral 64-slice CT scan were initially performed for PET attenuation correction and anatomical localization. The CT scans were acquired using a tube voltage of 120 kV in helical mode with Smart/Auto mA (range: 40–120 mA). The X-ray tube rotation time was set to 0.9 seconds, with a pitch of 0.984:1 and a table speed of 39.37 mm/rot. The helical thickness was 3.75 mm, and the standard reconstruction slice thickness was 2.5 mm. The GE ASIR (Adaptive Statistical Iterative Reconstruction) at 20% was utilized to minimize patient radiation dose from CT scans. Following CT, PET images were acquired in three dimensions from the vertex to mid-thigh for ^68^Ga-PSMA-11 scans and from the skull base to mid-thigh for ^18^F-FDG scans. Each bed position (15.7 cm with 23% overlap) had an acquisition time of 3 minutes. Emission data were corrected for geometric response, detector efficiency, system dead time, random coincidences, scatter, and attenuation.

Attenuation-corrected images were reconstructed using two vendor-provided iterative algorithms: OSEM and BPL. OSEM reconstruction settings were two iterations/24 subsets with a filter cut-off of 6.4 mm and a matrix size of 192 × 192. TOF (GE VUE Point FX) and a resolution recovery algorithm (GE SharpIR) were enabled. BPL images were reconstructed using the manufacturer’s default settings, which included a beta value of 350, TOF enabled, and no post-filtering. These settings were reviewed and confirmed as appropriate for clinical application by a medical physicist (R.K.) at our institution. The selected β value of 350 is also supported by previous literature as an optimal balance between image noise and lesion detectability in oncologic PET imaging (18–20). The BPL algorithm inherently includes PSF modeling with GE SharpIR automatically enabled.

### 2.3 Image analysis

Demographic and clinicopathologic data were collected, and the interpreters were aware of the clinical indications for the PET/CT scans. Image analysis was performed using the Hermes Workstation (Nuclear Diagnostics, Sweden). Each scan was independently evaluated in both OSEM and BPL reconstructions by two board-certified nuclear medicine physicians who were blinded to the reconstruction methods. In cases of discrepancies, a consensus was reached following a comprehensive discussion. All tracer-avid lesions with uptake higher than the background and not associated with physiological radiotracer uptake were considered malignant and reported. Lesions were recorded in both reconstruction methods. For semi-quantitative assessment, a region of interest (ROI) was drawn around the entire lesion on the axial PET image. For each lesion, the maximum and peak standardized uptake values (SUVmax and SUVpeak), as well as the diameter, were measured. Tumor-to-background ratios (TBR) were calculated by dividing the SUVmax of the lesion by the SUVmean of the background tissue. The liver background was used for liver lesions, mediastinal blood pool background for lymph nodes and peritoneal lesions, lung background for lung lesions, L5 background for bone lesions, and gluteal muscle for other lesions in ^18^F-FDG scans, and pelvic fat background for ^68^Ga-PSMA-11 PET scans. The intensity of uptake in each lesion was visually assessed for both reconstruction methods using a five-point scale similar to the Deauville five-point scale. In this scale, 1 indicates no uptake, 2 indicates uptake equal to or below the blood pool, 3 indicates uptake above the blood pool but below or equal to the liver uptake, 4 indicates uptake slightly to moderately higher than the liver, and 5 indicates markedly increased uptake. Volume measurements were obtained from CT scans using a semi-automated segmentation algorithm (21). ROIs were delineated on the CT images based on pre-defined threshold criteria, and manual adjustments were made by experienced nuclear medicine clinicians to ensure accuracy. Additionally, the mean and standard deviation (SD) of hepatic activity within a 3-cm spherical volume of interest centered in the right liver lobe, as well as the mean and SD of urinary bladder activity within a 3-cm spherical volume of interest centered in the urinary bladder, were recorded as SUVs.

### 2.4 Statistical analysis

Statistical analyses were conducted to compare the BPL and OSEM image reconstruction methods across various metrics, including lesion SUVmax, lesion SUVpeak, background SUVpeak, background SUVmean, liver SUVpeak, liver SUVmean, SD of liver SUVmean, bladder SUVmean, SD of bladder SUVmean, and TBR for both FDG and PSMA groups separately. The distribution of each metric was assessed for normality using the Shapiro-Wilk test. Due to the non-normal distribution of the data, non-parametric statistical tests were employed. The Wilcoxon signed-rank test was utilized to compare the metrics between BPL and OSEM reconstruction methods within each group (FDG and PSMA). Pearson correlation coefficients (r) were calculated to evaluate the linear relationship between the metrics obtained from BPL and OSEM reconstruction methods within each group. Additionally, the relationship between lesion volume and SUVmax was analyzed using Pearson correlation coefficients and linear regression analyses. Scatter plots with regression lines were generated to visually depict these relationships. Bland-Altman plots were generated to assess the agreement between the BPL and OSEM methods. These plots include the mean difference (bias) and the limits of agreement defined as the mean difference ± 1.96 standard deviations. All statistical analyses were conducted using R software (version 4.0.2). A p-value of <0.05 was considered statistically significant​​.

## 3 Results

Thirty-three patients (median age: 69 years, range: 38-87) with various malignancies were included in the study. Of these, 25 underwent ^18^F-FDG PET/CT (FDG group), and 8 underwent ^68^Ga-PSMA-11 PET/CT (PSMA group). A total of 100 lesions were identified: 80 in the FDG group and 20 in the PSMA group. Lesions were distributed in the lungs (12%), bones (12%), lymph nodes (52%), liver (4%), and other organs (20%). In the FDG group, the median lesion volume was 1.2 ml (IQR: 6.62 ml, range: 0.05 - 15.01 ml). In the PSMA group, the median lesion volume was 0.22 ml (IQR: 0.42 ml, range: 0.01 - 3.77 ml). Patient characteristics are presented in **Table 1**.

**Table 1:** Cohort characteristics.

Characteristic	Entire cohort (n=33, 100 lesions)	FDG group (n=25, 80 lesions)	PSMA group (n=8, 20 lesions)
Age (years), median (range)	69 (38-87)	66 (38-87)	73 (67-79)
Sex			
Female (%)	13 (39.3%)	13 (52%)	0 (0%)
Male (%)	20 (60.6%)	12 (48%)	8 (100%)
Weight (kg), median (range)	77 (49-117)	75 (49-117)	91 (71-110)
Height (cm), median (range)	169 (150-188)	165 (150-188)	172 (167-182)
Injected activity (MBq), median (range)	338 (239-446)	368 (239-446)	334 (322-347)
Uptake time (min), median (range)	60 (57-64)	59 (57-64)	61 (58-64)
Type of cancer			
Prostate cancer	8 (24.2%)	0 (0%)	8 (100%)
Lung cancer	7 (21.2%)	7 (28.0%)	–
Head and neck SCC	3 (9.1%)	3 (12.0%)	–
Lymphoma	8 (24.2%)	8 (32.0%)	–
Cervical cancer	2 (6.1%)	2 (8.0%)	–
Melanoma	2 (6.1%)	2 (8.0%)	–
Colon cancer	1 (3.0%)	1 (4.0%)	–
Esophageal cancer	2 (6.1%)	2 (8.0%)	–
Number of lesions			
1	6 (18.2%)	4 (16.0%)	2 (25.0%)
2	10 (30.3%)	7 (28.0%)	3 (37.5%)
3	10 (30.3%)	8 (32.0%)	2 (25.0%)
4	2 (6.1%)	2 (8.0%)	0 (0%)
5	1 (3.0%)	1 (4.0%)	0 (0%)
>5	4 (12.1%)	3 (12.0%)	1 (12.5%)
Site of lesions			
Bone	12 (12%)	4 (5.0%)	8 (40.0%)
Lymph node	52 (52%)	43 (53.7%)	9 (45.0%)
Lung	12 (12%)	10 (12.5%)	2 (10.0%)
Liver	4 (4.0%)	4 (5.0%)	0 (0%)
Other	20 (20%)	19 (23.7%)	1 (5.0%)

SCC, Squamous cell carcinoma; FDG, Fluorodeoxyglucose; PSMA, Prostate-Specific Membrane Antigen.

**Figure 1:**
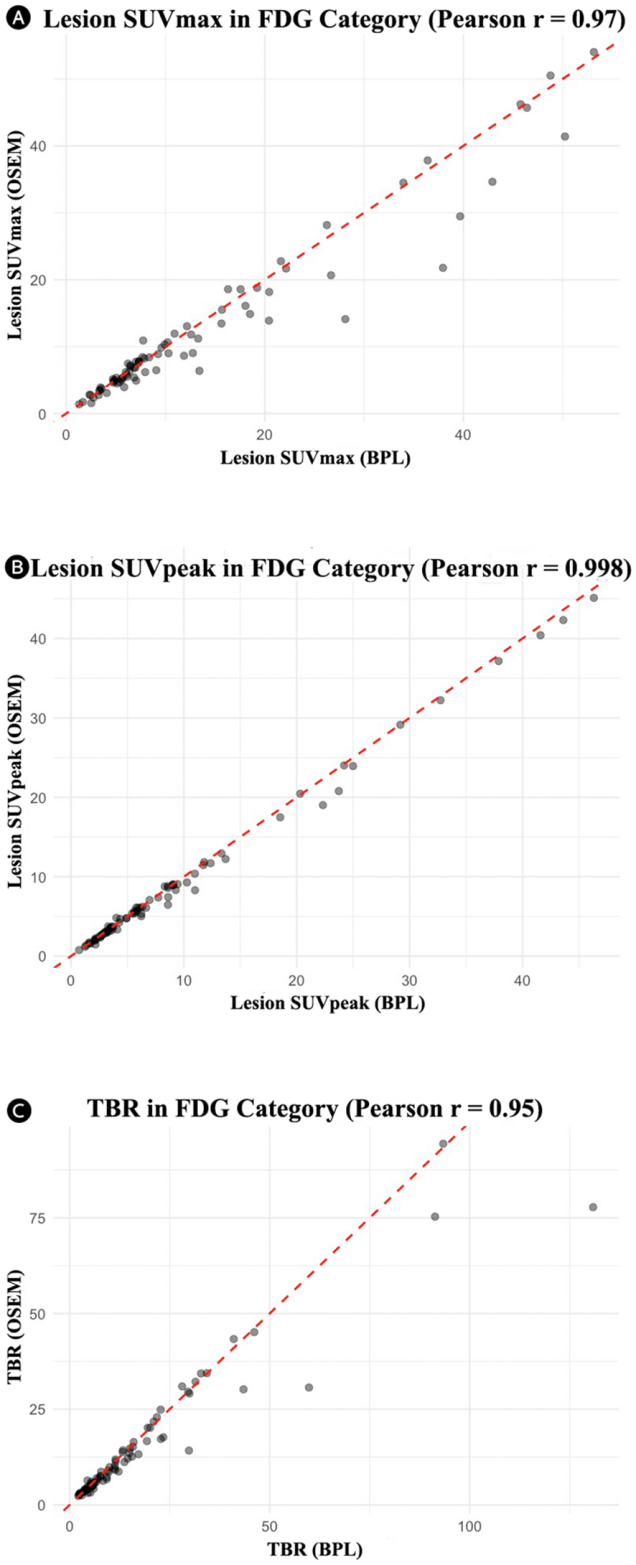
Correlation plots for the FDG group showing the relationship between OSEM and BPL metrics: **(A)** lesion SUVmax (r = 0.97), **(B)** lesion SUVpeak (r = 0.998), and **(C)** tumor-to-background ratio (r = 0.95). In all cases, the line of best fit did not significantly differ from the unity slope. FDG, Fluorodeoxyglucose; OSEM, Ordered Subsets Expectation Maximization; SUV, Standardized Uptake Value; TBR, Tumor-to-Background Ratio. BPL corresponds to Q.Clear (GE Healthcare).

**Figure 2:**
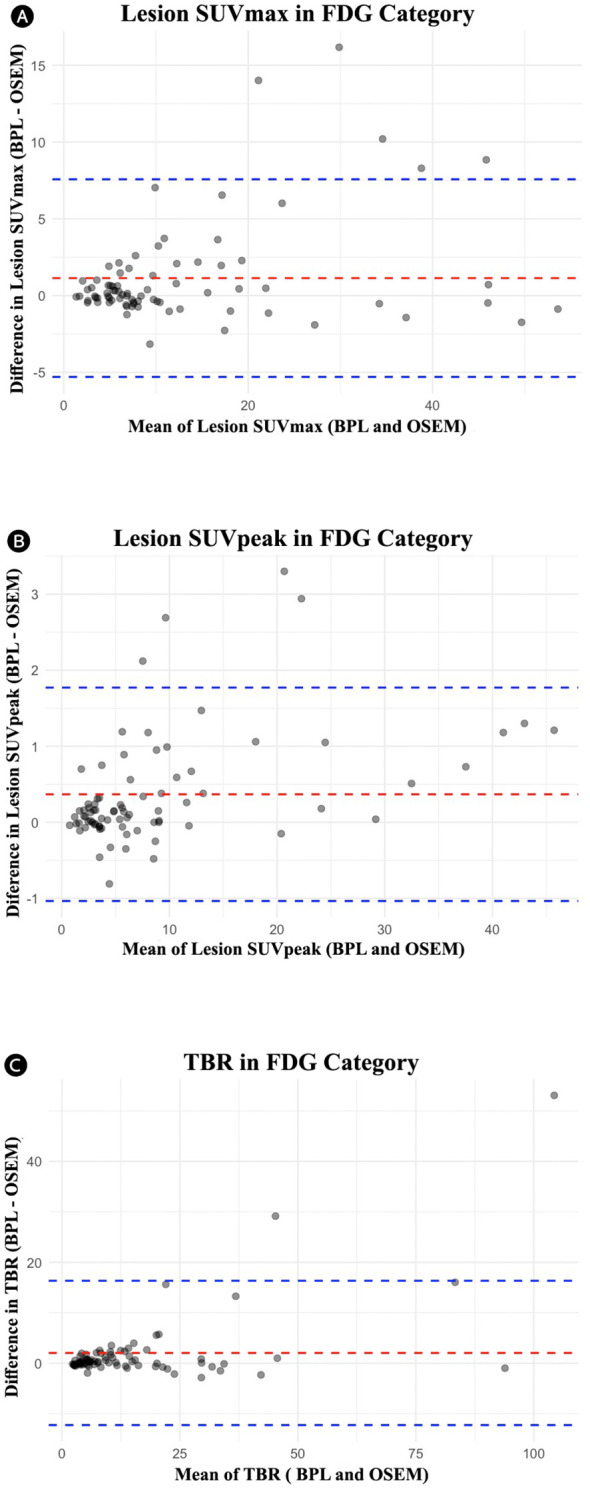
Bland-Altman plots for the FDG group comparing OSEM and BPL for various metrics: **(A)** lesion SUVmax (mean difference: 1.14, limits of agreement: -5.30 to 7.57), **(B)** lesion SUVpeak (mean difference: 0.37, limits of agreement: -1.04 to 1.77), and **(B)** tumor-to-background ratio (TBR) (mean difference: 2.04, limits of agreement: -12.26 to 16.34). FDG, Fluorodeoxyglucose; OSEM, Ordered Subsets Expectation Maximization; BPL, Bayesian Penalized Likelihood; SUV, Standardized Uptake Value; TBR, Tumor-to-Background Ratio. BPL corresponds to Q.Clear (GE Healthcare).

**Table 2** summarizes the comparison between OSEM and BPL image reconstruction methods across various metrics for FDG and PSMA categories. Significant differences between the methods were observed in several metrics. For the FDG group, significant differences were found in lesion SUVpeak, liver SUVpeak, SD of liver SUVmean, bladder SUVmean, SD of bladder SUVmean, and TBR, with BPL generally producing higher values except for liver SUVpeak, SD of liver SUVmean, and SD of bladder SUVmean, where OSEM reconstructed images showed higher values. The Pearson correlation coefficients were high for all metrics, indicating strong positive correlations between BPL and OSEM (**Figure 1**). Furthermore, the slope of these lines did not significantly differ from 1, indicating good agreement between measurements. The Bland-Altman plots (**Figure 2**), however, indicate average differences (red lines) that are statistically significantly different from 0. This corresponds to the p-values <0.05 in **Table 2** for the same metrics.

**Table 2:** Comparison of OSEM and BPL image reconstruction methods across various metrics for FDG and PSMA categories.

Metric	Category	OSEM median (IQR)	BPL median (IQR)	Wilcoxon V	P-value	Pearson r	Mean difference	Limits of agreement	95% Confidence interval
Lesion SUVmax	**FDG**	**7.81 (4.84-9.91)**	**7.82 (4.88-12.11)**	**2028.5**	**0.050**	**0.97**	**1.14**	**-5.30 to 7.57**	**0.41 to 1.87**
	**PSMA**	**6.25 (3.50-5.68)**	**7.50 (4.66-11.79)**	**182.0**	**0.003**	**0.93**	**2.98**	**-5.65 to 11.62**	**0.92 to 5.04**
Lesion SUVpeak	**FDG**	**5.44 (3.20-5.97)**	**5.74 (3.28-6.48)**	**2423.0**	**0.0006**	**0.998**	**0.37**	**-1.04 to 1.77**	**0.21 to 0.53**
	PSMA	3.50 (2.20-2.29)	3.58 (2.28-2.68)	139.5	0.20	0.99	0.41	-1.37 to 2.18	-0.02 to 0.83
Background SUVpeak	FDG	1.07 (0.73-1.83)	1.06 (0.72-1.80)	726.0	0.08	0.99	-0.01	-0.13 to 0.11	-0.03 to -0.00
	PSMA	0.81 (0.55-1.16)	0.76 (0.56-1.05)	53.5	0.17	0.99	-0.02	-0.15 to 0.10	-0.05 to 0.01
Background SUVmean	FDG	0.94 (0.67-1.73)	0.94 (0.69-1.75)	710.5	0.09	0.99	0.00	-0.07 to 0.07	-0.01 to 0.00
	PSMA	0.72 (0.54-1.02)	0.67 (0.55-0.98)	65.0	0.38	0.98	-0.02	-0.18 to 0.13	-0.06 to 0.01
Liver SUVpeak	**FDG**	**2.89 (2.43-3.25)**	**2.75 (2.38-3.13)**	**0.0**	**0.0008**	**0.99**	**-0.10**	**-0.25 to 0.04**	**-0.12 to -0.09**
	**PSMA**	**6.14 (5.42-6.84)**	**6.15 (5.47-6.70)**	**21.0**	**0.002**	**0.999**	**-0.17**	**-0.50 to 0.16**	**-0.25 to -0.09**
Liver SUVmean	FDG	2.37 (2.13-2.65)	2.38 (2.12-2.67)	859.0	0.62	0.98	-0.01	-0.19 to 0.16	-0.03 to 0.01
	PSMA	5.63 (5.18-6.44)	5.67 (5.30-6.61)	98.0	0.31	0.99	0.02	-0.18 to 0.23	-0.02 to 0.07
SD of the Liver SUVmean	**FDG**	**0.36 (0.21-0.34)**	**0.22 (0.14-0.27)**	**0.0**	**0.0007**	**0.63**	**-0.12**	**-0.24 to -0.01**	**-0.14 to -0.11**
	**PSMA**	**0.49 (0.35-0.59)**	**0.37 (0.29-0.46)**	**0.0**	**0.0009**	**0.96**	**-0.14**	**-0.26 to -0.02**	**-0.17 to -0.11**
Bladder SUVmean	**FDG**	**27.91 (14.99-42.60)**	**28.81 (15.03-43.12)**	**2850.0**	**0.0008**	**0.99**	**2.23**	**-9.79 to 14.25**	**0.87 to 3.60**
	PSMA	14.59 (10.91-16.11)	14.70 (11.00-16.75)	107.0	0.95	0.05	77.71	-603.99 to 759.42	-85.07 to 240.49
SD of Bladder mean	**FDG**	**1.53 (1.08-2.77)**	**1.25 (0.87-2.54)**	**1037.0**	**0.005**	**0.998**	**-0.28**	**-2.75 to 2.20**	**-0.56 to 0.00**
	**PSMA**	**0.71 (0.56-1.16)**	**0.65 (0.44-0.65)**	**39.0**	**0.01**	**0.94**	**-0.09**	**-0.60 to 0.42**	**-0.21 to 0.03**
TBR	**FDG**	**8.70 (4.55-12.02)**	**9.66 (5.50-14.56)**	**2220.0**	**0.004**	**0.95**	**2.04**	**-12.26 to 16.34**	**0.42 to 3.66**
	**PSMA**	**8.31 (4.32-9.42)**	**8.83 (4.77-20.06)**	**178.0**	**0.005**	**0.86**	**5.45**	**-13.00 to 23.89**	**1.04 to 9.85**

OSEM, Ordered Subset Expectation Maximization; BPL, Bayesian Penalized Likelihood; FDG, 18F-Fluorodeoxyglucose; PSMA, Prostate-Specific Membrane Antigen; SUVmax, Maximum Standardized Uptake Value; SUVpeak, Peak Standardized Uptake Value; SUVmean, Mean Standardized Uptake Value; SD, Standard Deviation; TBR, Tumor-to-Background Ratio; IQR, Interquartile Range. BPL corresponds to Q.Clear (GE Healthcare). P-value<0.05 is statistically significant.

**Figure 3:**
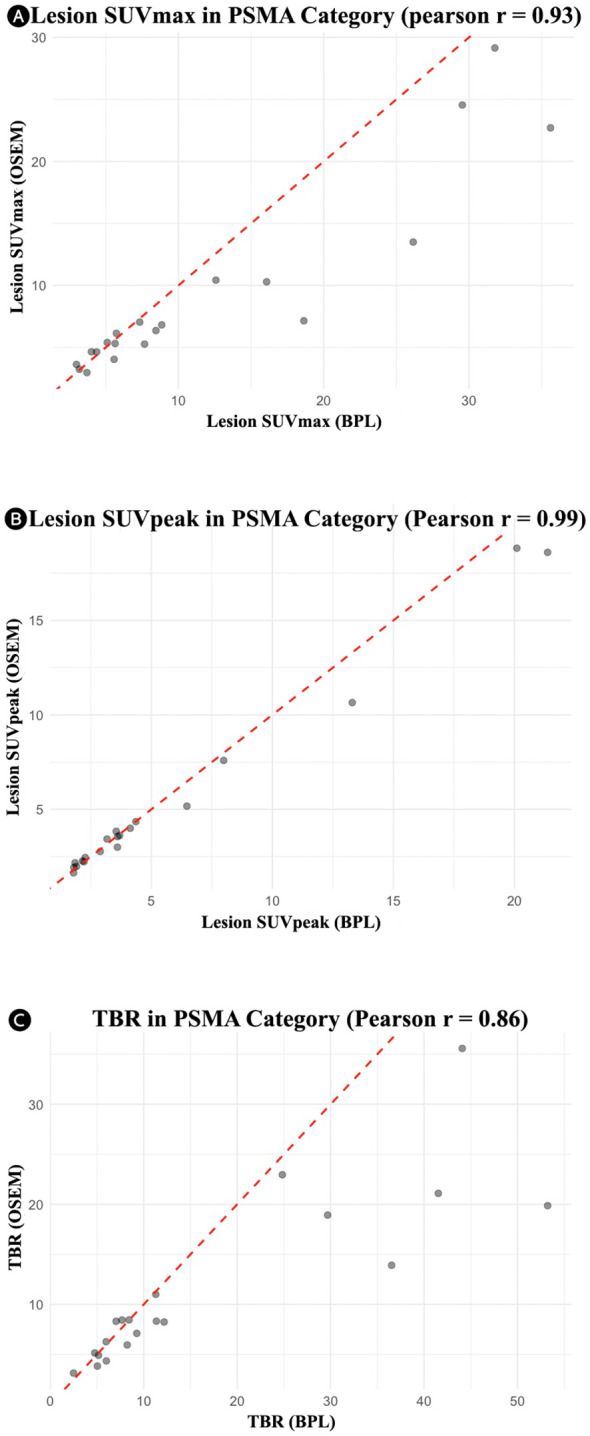
Correlation plots for the PSMA group showing the relationship between OSEM and BPL metrics: **(A)** lesion SUVmax (r = 0.93), **(B)** lesion SUVpeak (r = 0.99), and **(C)** tumor-to-background ratio (r = 0.86). PSMA, Prostate-Specific Membrane Antigen; OSEM, Ordered Subsets Expectation Maximization; BPL, Bayesian Penalized Likelihood; SUV, Standardized Uptake Value; TBR, Tumor-to-Background Ratio. BPL corresponds to Q.Clear (GE Healthcare).

**Figure 4:**
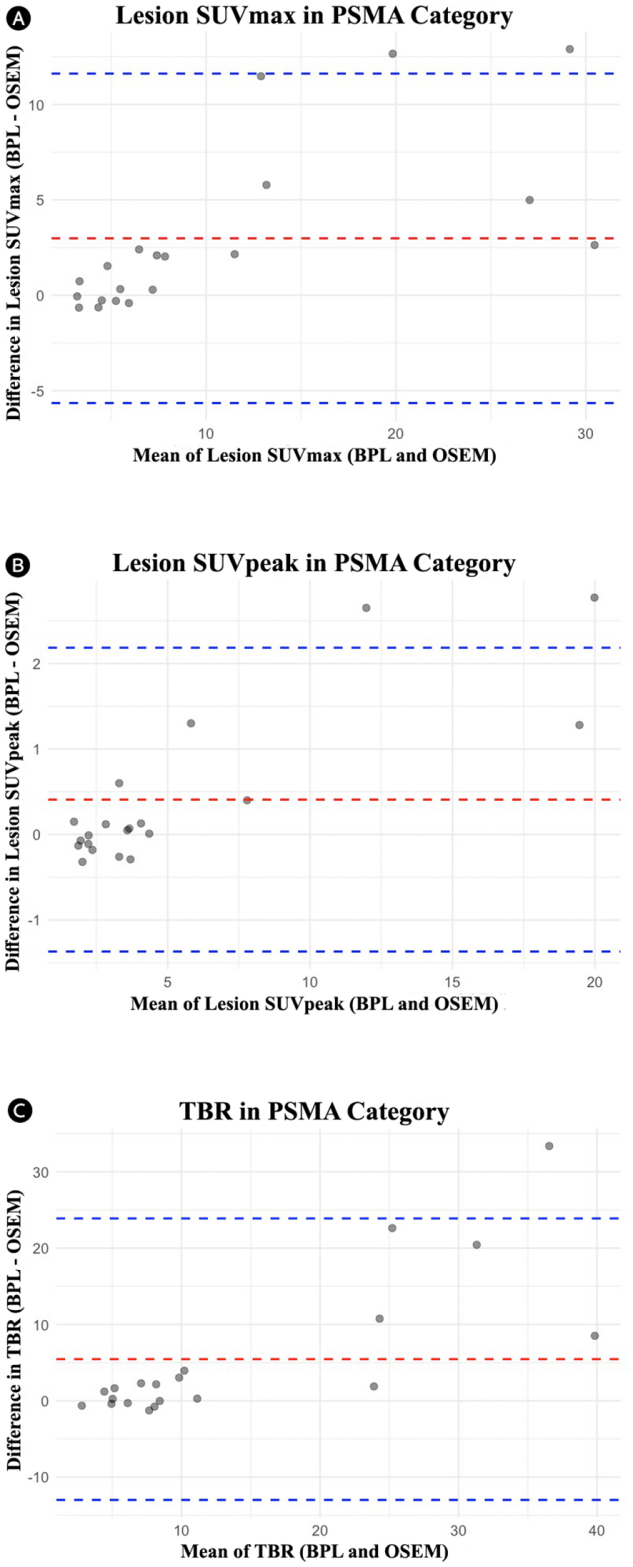
Bland-Altman plots for the PSMA group comparing OSEM and BPL for various metrics: **(A)** lesion SUVmax (mean difference: 2.98, limits of agreement: -5.65 to 11.62), **(B)** lesion SUVpeak (mean difference: 0.41, limits of agreement: -1.37 to 2.18), and **(C)** tumor-to-background ratio (mean difference: 5.45, limits of agreement: -13.00 to 23.89). PSMA, Prostate-Specific Membrane Antigen; OSEM, Ordered Subsets Expectation Maximization; BPL, Bayesian Penalized Likelihood; SUV, Standardized Uptake Value; TBR, Tumor-to-Background Ratio. BPL corresponds to Q.Clear (GE Healthcare).

For the PSMA group, significant differences were observed in lesion SUVmax, liver SUVpeak, SD of liver SUVmean, SD of bladder SUVmean, and TBR, with BPL reconstruction enhancing all these metrics except for SD of liver SUVmean and SD of bladder SUVmean, where OSEM reconstruction resulted in higher values. The Pearson correlation coefficients were also high for most metrics, suggesting strong correlations between the two methods (**Figure 3**). Bland-Altman plots (**Figure 4**) illustrated the agreement between BPL and OSEM, showing generally good correlation with some variability in specific metrics.

In the FDG group, the median visual intensity scores were 3 for both BPL and OSEM. For the PSMA group, the median visual intensity scores were 4 for BPL and 3 for OSEM. The Wilcoxon signed-rank test did not show a statistically significant difference between the two methods.

**Figure 5:**
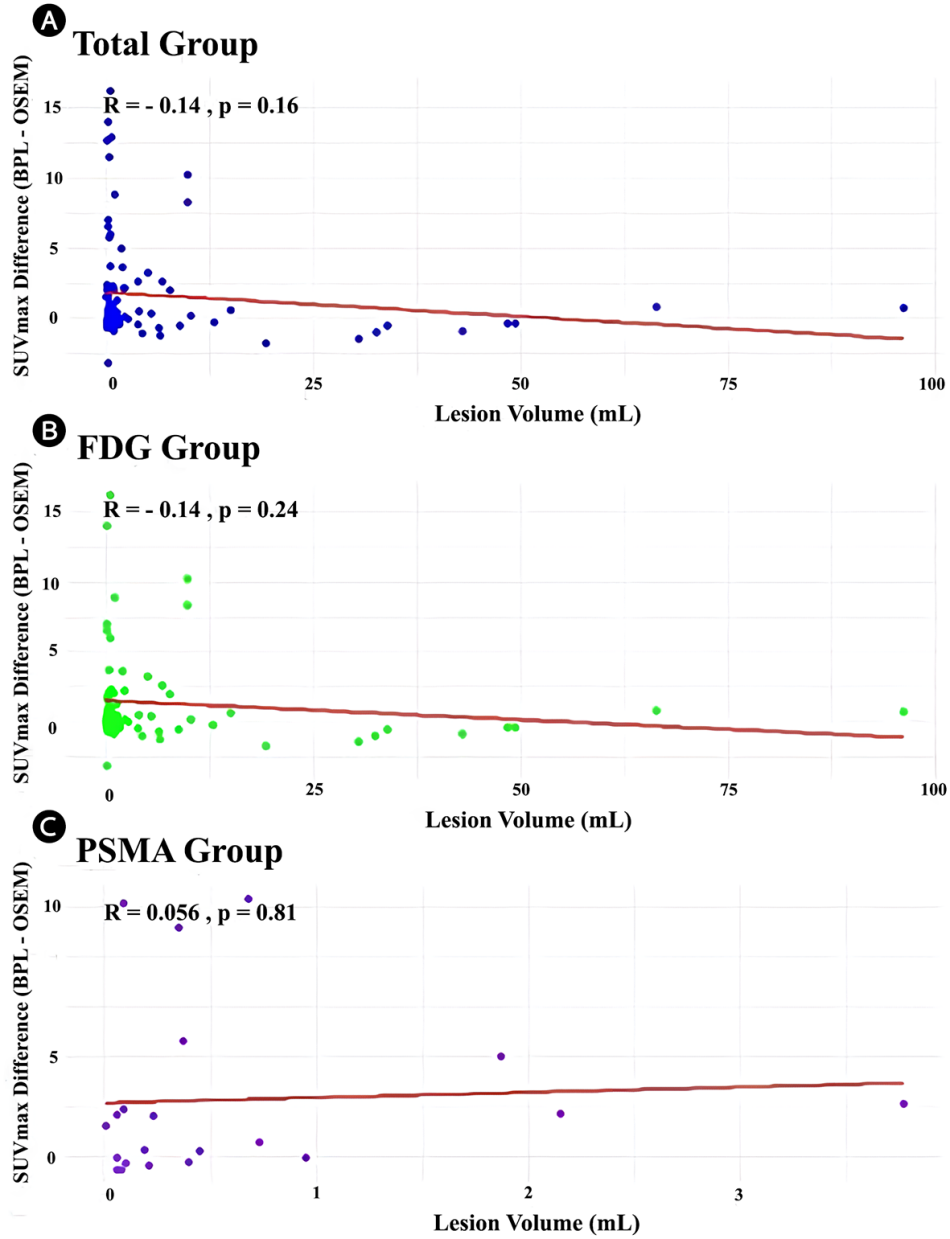
Scatter plots demonstrating the relationship between lesion volume (mL) and SUVmax difference (BPL [Q.Clear] – OSEM) for the **(A)** total cohort, **(B)** FDG group, and **(C)** PSMA group. Linear regression lines (red) are displayed with the corresponding Pearson correlation coefficients (r) and p-values. (r) and corresponding p-values are displayed on each panel. FDG, Fluorodeoxyglucose; PSMA, Prostate-Specific Membrane Antigen; OSEM, Ordered Subsets Expectation Maximization; BPL, Bayesian Penalized Likelihood; SUV, Standardized Uptake Value. BPL corresponds to Q.Clear (GE Healthcare), P-value<0.05 is statistically significant.

The relationship between lesion volume and SUVmax differences (BPL– OSEM) was analyzed for the total cohort and stratified by tracer groups (FDG and PSMA). In the total cohort, a weak negative correlation was observed between lesion volume and SUVmax difference (r = -0.14, p = 0.16), although this relationship was not statistically significant. Similarly, in the FDG group, a weak negative correlation was noted (r = -0.14, p = 0.24), which also did not reach statistical significance. Scatter plots with linear regression lines illustrate the distribution of data (**Figure 5**).

**Figure 6:**
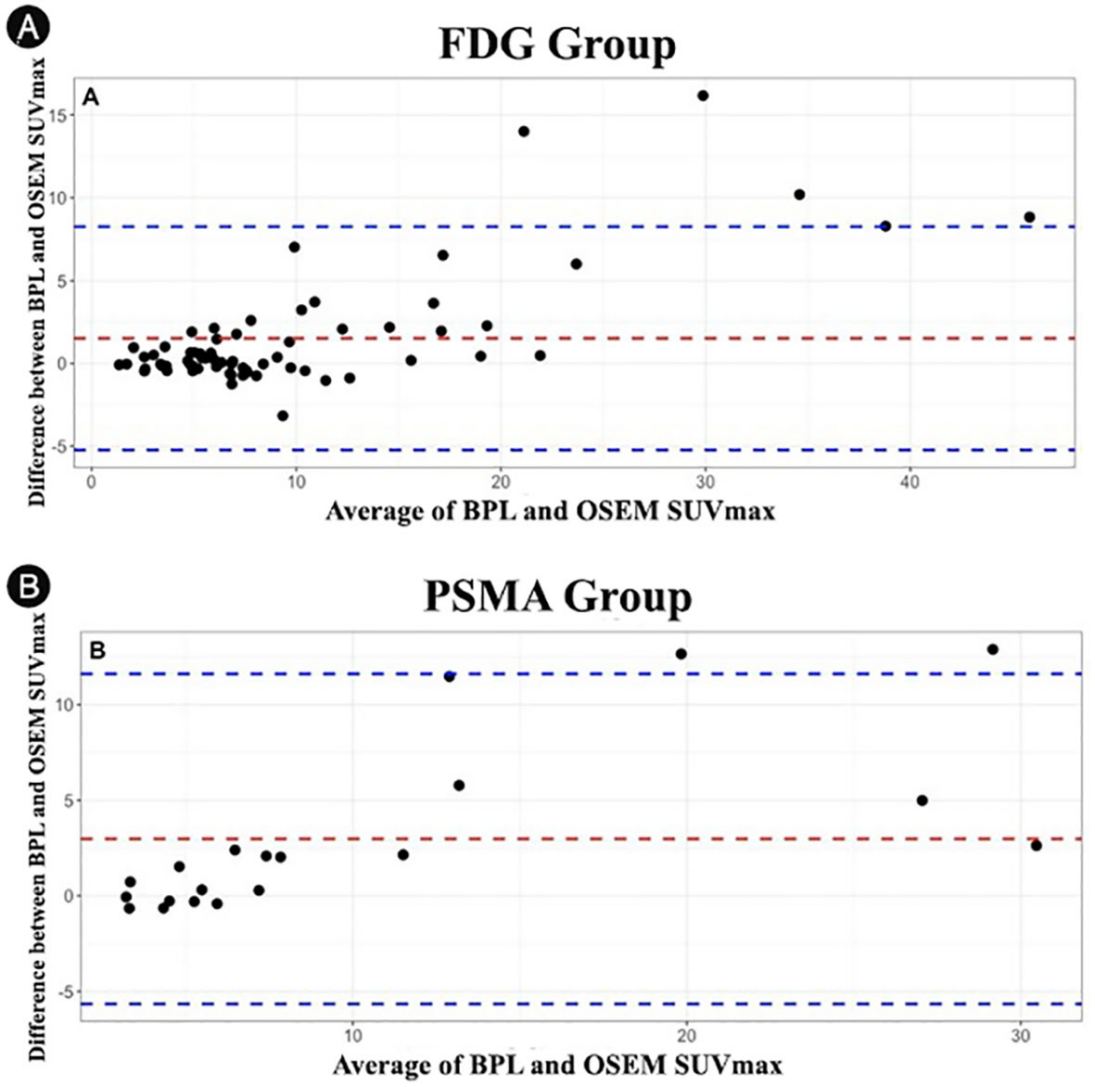
Bland-Altman plots showing the differences in SUVmax values between OSEM and BPL reconstruction methods for different lesion volumes: **(A)** FDG group and **(B)** PSMA group. FDG, Fluorodeoxyglucose; PSMA, Prostate-Specific Membrane Antigen; OSEM, Ordered Subsets Expectation Maximization; BPL, Bayesian Penalized Likelihood; SUV, Standardized Uptake Value. BPL corresponds to Q.Clear (GE Healthcare).

The differences in SUVmax values between BPL and OSEM reconstruction methods were analyzed using Bland-Altman plots (**Figure 6**). The Bland-Altman plots showed that the differences in SUVmax were more pronounced for higher average SUVmax values (calculated as the mean of BPL and OSEM SUVmax) in both the FDG and PSMA groups. The mean bias was positive in both groups, indicating that BPL consistently yielded higher SUVmax values compared to OSEM. However, the limits of agreement were wider for lesions with higher SUVmax, suggesting greater variability between the two reconstruction methods in these cases. These findings highlight that while the reconstruction methods generally agree, BPL may yield higher SUVmax values than OSEM, particularly for lesions with elevated SUVmax.

**Figure 7:**
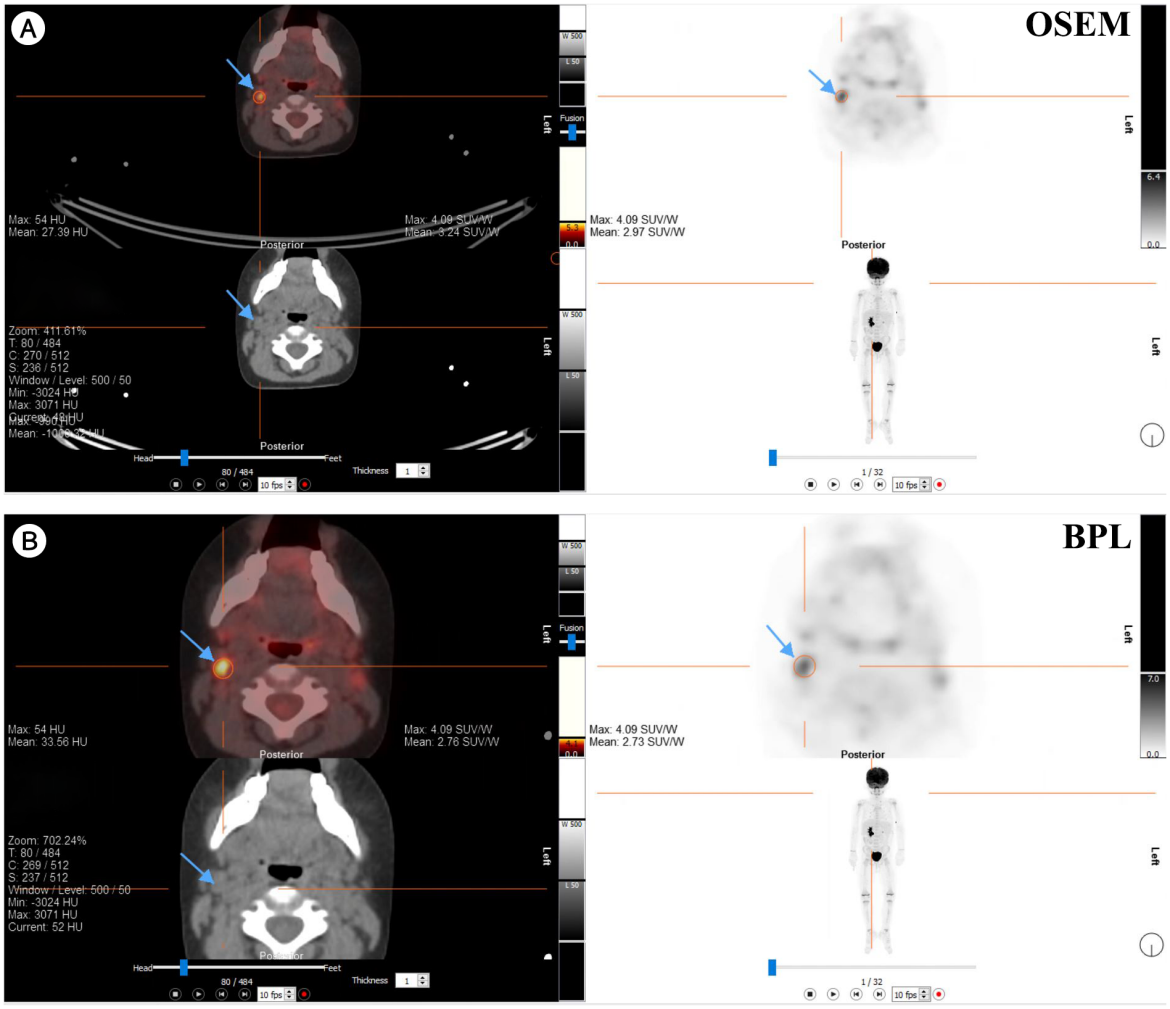
Comparison of PET/CT reconstruction results using OSEM and BPL algorithms with ^18^F-FDG in a patient with a right neck Level IIA lymph node mass. **(A)** OSEM reconstruction; **(B)** BPL reconstruction. FDG, Fluorodeoxyglucose; OSEM, Ordered Subsets Expectation Maximization; BPL, Bayesian Penalized Likelihood; SUV, Standardized Uptake Value. BPL corresponds to Q.Clear (GE Healthcare).

**Figure 8:**
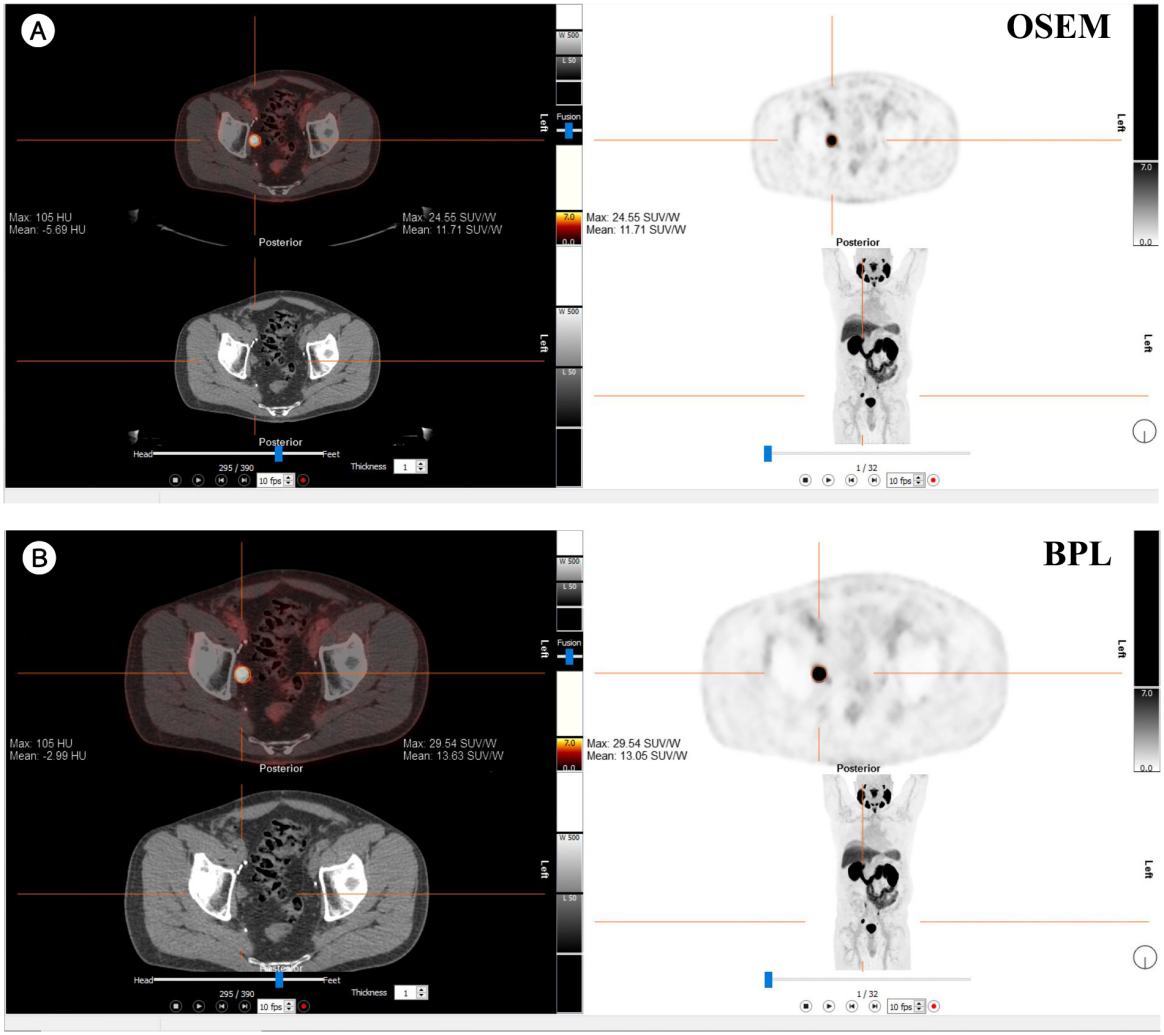
Comparison of PET/CT reconstruction results using OSEM and BPL algorithms with ^68^Ga-PSMA-11 in a patient with a right external iliac mass. **(A)** OSEM reconstruction; **(B)** BPL reconstruction. FDG, Fluorodeoxyglucose; OSEM, Ordered Subsets Expectation Maximization; BPL, Bayesian Penalized Likelihood; SUV, Standardized Uptake Value; BPL corresponds to Q.Clear (GE Healthcare).

**Figures 7**, **8** present representative PET/CT images using OSEM and BPL reconstruction methods for ^18^F-FDG and ^68^Ga-PSMA-11, respectively, highlighting differences in image quality and lesion visualization.

## 4 Discussion

BPL reconstruction, also known as Q.Clear in GE Healthcare Imaging, is a relatively new method that enhances contrast over OSEM by applying a noise penalty to individual voxels during reconstruction (7, 8). From a clinical perspective, BPL reconstruction improves the signal-to-noise ratio (SNR) and has been reported to be useful for better localization of tumors, as evidenced in case reports (22). However, variations in reconstruction methods on the same PET study can lead to downstream consequences, including variations in biomarkers and radiomics features derived from the PET study (23).

Several studies have reported superior overall image quality with BPL (Q.Clear) compared to OSEM in various PET/CT scans, including ^68^Ga-DOTANOC (24), ^18^F-fluciclovine (13), ^68^Ga-PSMA (9), and ^18^F-FDG scans (15). This improvement may be attributed to the inability of OSEM reconstruction to achieve full convergence due to increased noise and iteration times. However, further studies are necessary to determine the optimal β value for reconstructions that ensure the best image quality (7, 13, 24). The BPL reconstruction with β = 350 was implemented in this study based on three key considerations: 1) manufacturer recommendations for clinical oncologic PET imaging, 2) formal validation by our institutional medical physicist (R.K.), and 3) robust literature evidence (18–20). Prior studies suggest β values significantly affect quantification, particularly in small lesions (8, 9). Texte et al. (18) identified β = 350 as optimal for contrast recovery in low-contrast hypoxic imaging (SBR = 3), reporting a 27.89% improvement in contrast recovery coefficient (CRC) over OSEM without compromising detectability—findings particularly relevant to our study of small PSMA-positive lesions (median volume: 0.22 mL). Similarly, other investigations demonstrated that intermediate β-values (350–400) offer the best trade-off between noise suppression and quantification accuracy by applying relative difference penalties during iterative reconstruction (19). Contemporary studies further support this parameter range, indicating that lower β-values (e.g., 200) can excessively amplify SUVmax in sub-centimeter lesions, while higher values (500–1000) tend to over-suppress lesion conspicuity (25). Our findings indicate that β = 350 improves SUVmax and TBR compared to OSEM, while preserving diagnostic interpretability across lesion sizes. Notably, this parameter selection aligns with EARL harmonization standards, as the observed <10% inter-method variation falls well within established accreditation tolerances (26). The enhanced image quality provided by the BPL algorithm can be explained by its ability to reduce noise through an adaptive filter with adjustable filter width and improve contrast by increasing quantification, thereby creating the effect of better image quality.

Although our study found statistically significant increases in quantitative PET metrics (SUVmax: +1.14 for FDG, +2.98 for PSMA; SUVpeak: +0.37 for FDG, +0.41 for PSMA) with BPL reconstruction relative to OSEM, these variations were well within the limits of accreditation variability established (27). While Bland-Altman limits of agreement (-5.30 to +7.57 for FDG SUVmax) are consistent with the Quantitative Imaging Biomarkers Alliance (QIBA) Profile’s approximately 30% repeatability coefficient for non-harmonized protocols (28), the <10% inter-method variation aligns with the European Association of Nuclear Medicine Research Ltd (EARL) harmonization program’s 10% calibration bias tolerance for accredited systems (26). Except for QIBA’s 28–39% threshold for clinically significant biological variation, all observed differences correspond to slightly higher variability for tiny PSMA lesions (median lesion volume 0.22 mL) and EARL’s 20% recovery coefficient allowance for sub-10 mm spheres (29). These results confirm large-scale harmonization data from more than 200 EARL-accredited systems (post-correction SD: 3.7%) and that BPL-derived increments, while statistically significant, are improbable to affect clinical interpretation or PET Response Criteria in Solid Tumors (PERCIST), given reconstruction techniques stay consistent for longitudinal studies (27, 30).

This study aimed to evaluate and compare the OSEM algorithm with the BPL Q.Clear algorithm for PET/CT image reconstruction using ^18^F-FDG and ^68^Ga-PSMA-11 tracers. Our analysis focused on the impact of these reconstruction methods on key quantitative metrics such as SUVmax, SUVpeak, background SUV, and TBR. A key strength of this study is the application of these two reconstruction methods to the same set of patients, allowing for a direct comparison of images with identical characteristics except for the reconstruction method. Our results indicate significant differences between the BPL and OSEM algorithms across several quantitative metrics. In the FDG group, BPL generally produced higher values for lesion SUVpeak, bladder SUVmean, and TBR but lower values for liver SUVpeak and the SD of liver and bladder SUVmean compared to OSEM. These differences were also observed in the PSMA group, where BPL resulted in higher lesion SUVmax, liver SUVpeak, and TBR values but lower SDs for liver and bladder SUVmean. Recent studies have reported that BPL reconstruction produces higher SUVmax values than OSEM in PET imaging, especially for smaller lesions (14, 25, 31, 32). SUVmax-based analyses have shown that BPL increases SUVmax in lesions with low SUVmax (<5) in OSEM images while decreasing it in those with high SUVmax (>10). A study comparing different beta values found that BPL with a beta of 200 results in significantly higher tumor SUVmax than OSEM, whereas beta values of 400, 500, or 1000 lead to lower SUVmax. In small lesions (≤2 cm), the percentage difference in SUVmax between OSEM and BPL (beta 200) and between BPL (beta 200) and BPL (beta 1000) was greater than in larger lesions, indicating that BPL enhances image quality while maintaining accurate quantification, with beta 500 identified as the most suitable (25). Investigations in lymphoma have examined the impact of reconstruction algorithms on quantitative evaluation, highlighting the influence of lymph node size (32). Measurement differences were more pronounced in small lesions, with BPL affecting SUVmax more than SUVmean or SUVpeak, suggesting potential implications for Deauville score assessment and patient management (32). Despite these variations, strong correlations between BPL- and OSEM-derived quantitative parameters in ^68^Ga-PSMA-11 PET/CT have been reported, indicating that both methods provide consistent results without significant clinical impact on quantitative or volumetric findings (14). Similarly, in our study, both reconstruction methods demonstrated high Pearson correlation coefficients across most metrics, indicating strong positive correlations between BPL and OSEM. This suggests that while absolute values may differ, the relative rankings of lesion uptake are preserved between the methods, which is crucial for consistent patient management and follow-up studies. In the present study, uniform regions such as liver, bladder, and background demonstrated small differences that may not be clinically significant (**Table 2**). Furthermore, good agreement between BPL and OSEM was noted in large lesions (**Figure 5**). However, this figure also shows that the differences in SUVmax between the two methods become increasingly pronounced with smaller lesion volumes. Interestingly, regression analysis revealed that while the differences in SUVmax between BPL and OSEM showed weak or non-significant correlations with lesion volume for the FDG and PSMA groups, the overall trend indicates that these differences are more pronounced for smaller lesions. This suggests that BPL reconstruction may yield higher SUVmax values in small lesions compared to OSEM, highlighting its potential advantages in evaluating smaller lesions while maintaining good agreement in larger lesions. These observations may be partially explained by partial volume effects (PVE), which arise when the spatial resolution of PET imaging is insufficient to accurately quantify activity in small lesions. While partial volume correction (PVC) was not applied in this study, consistent with routine clinical workflows where such corrections are rarely implemented, the impact of PVE remains a relevant consideration. In OSEM, small lesions often suffer from underestimation of activity, whereas BPL has been shown to overestimate SUVmax in sub-centimeter lesions depending on β-value and lesion-to-background contrast (20, 33). To reduce the influence of PVE, we included SUVpeak as a secondary metric, which has been shown to be less sensitive to spatial resolution limitations and more reproducible across platforms (29, 34). The strong correlation between BPL- and OSEM-derived metrics (r > 0.9) supports the internal consistency of our data, although future studies incorporating standardized PVC methods may further refine quantification accuracy, especially in studies focused on small or sub-centimeter lesions.

A study found that BPL reconstructions produced notably higher SUVmax values for tumor lesions compared to standard OSEM reconstructions, with relatively greater increases seen in smaller-sized lesions (7). Similarly, Witkowska-Patena et al. showed that BPL SUVs and TBR are generally higher in ^18^F-PSMA-1007 PET/CT scans, particularly for small and highly avid lesions, without affecting the specificity and sensitivity of ^18^F-PSMA-1007 PET/CT (16). This effect is emphasized as an area requiring harmonization by the EARL FDG PET/CT accreditation program (17). According to the committee, reconstruction techniques that enhance the appearance of small lesions through additional processing may lead to inconsistent quantification of common PET biomarkers across different sites or equipment. While our study supports the observation of higher average SUVmax on BPL compared to OSEM reconstructed images, it also shows a strong correlation between SUVmax values produced by the two methods (Pearson r = 0.97 for FDG and r = 0.93 for PSMA). However, the wide limits of agreement observed in absolute values suggest that these methods may not be used interchangeably without appropriate harmonization or standardization in longitudinal studies. The correlation plots indicate that differences in SUVs become more pronounced at higher SUVs. Given the SNR recovery of the BPL method compared to OSEM, harmonizing SUVs should be feasible, as demonstrated in other studies (35). However, our study does not support significantly higher relative increases of SUVmax in smaller lesions. On the contrary, our results indicated higher relative increases in lesions with larger volumes. A recent study by Ayati et al. also showed that BPL algorithm reconstructions resulted in higher SUVmax and SUVmean and lower Metabolic Tumor Volume (MTV)-PSMA compared to the OSEM group, with a strong correlation between SUVmax, SUVmean, and MTV-PSMA values in OSEM and BPL reconstructed images (14).

Naghavi-Behzad et al. reported that images from patients with metastatic breast cancer showed better sharpness, contrast, higher SUVmax, and SULpeak using BPL reconstruction, while OSEM reconstruction had a less blotchy appearance (15). These results align with other studies indicating that BPL allows a significant increase in quantitative parameters (13, 18). Lundeberg et al. compared the two reconstruction modalities in lung cancer patients and found similar results, indicating that BPL reconstruction provides higher SUVmax for suspected lymph node metastases compared to OSEM reconstruction. However, higher SUVmax values did not improve the detection of metastatic lesions (36).

These findings have important implications for clinical practice. While both BPL and OSEM are effective for PET/CT imaging, the choice of reconstruction method can affect quantitative parameters, which should be considered when interpreting PET/CT results, especially in longitudinal studies. Consistent use of the same reconstruction method is recommended to ensure reliability and comparability of results over time. However, using identical acquisition and processing protocols may not always be feasible, particularly in referral centers where new patients with externally acquired baseline studies are frequently referred. For centers using BPL reconstruction, it is important to note that differences are more profound at higher SUVs and larger lesion volumes.

### 4.1 Limitations

Several limitations of our study should be noted. The retrospective design and the relatively small sample size may limit the generalizability of our findings. Although the total lesion count was 100 (80 FDG, 20 PSMA), the small number of lesions detected by ^68^Ga-PSMA-11 PET/CT may reduce the statistical power for subgroup analyses. This limitation could affect the precision and generalizability of tracer- or lesion-specific results, warranting cautious interpretation. Additionally, considering that the current study was conducted using a single β value for BPL reconstruction, further studies could be beneficial to explore the impact of varying β values across different radiotracers and clinical contexts to fully optimize the performance of the BPL algorithm. Moreover, PVC was not performed in this study, which may affect quantitative accuracy, particularly in small lesions. Although SUVpeak was included to reduce PVE-related bias, future studies incorporating standardized PVC techniques may provide more accurate quantification in sub-centimeter lesions. Besides, while this study focused on comparing the technical performance of OSEM and BPL reconstruction algorithms through quantitative metrics (SUVmax, TBR), we acknowledge the lack of clinical endpoint assessment as a limitation. As demonstrated in our results and supported by existing harmonization frameworks (26, 28, 30), the observed inter-method differences fell within variability thresholds unlikely to alter clinical interpretation. Nevertheless, prospective studies directly correlating BPL-driven quantitative enhancements with clinical outcomes (e.g., staging changes or treatment adaptation) remain necessary to validate their real-world impact. Furthermore, while lesion volumes were measured using a semi-automated CT-based segmentation algorithm and independently reviewed by two board-certified nuclear medicine physicians, only the consensus values were analyzed. Formal intra- and inter-observer reproducibility metrics were not assessed, limiting the quantitative evaluation of reader variability. Nevertheless, the consensus approach likely reduced individual variability. Prior studies, such as Gotra et al. (21), have demonstrated high reproducibility (ICC ≥ 0.99) for similar segmentation methods, supporting the robustness of our methodology. Future studies should include formal reproducibility assessments and validate these findings in larger, prospective cohorts and across multiple centers to enhance their robustness and generalizability. Exploring the optimal β values for BPL across various PET tracers and clinical conditions will be crucial. Comparative analyses involving newer PET/CT systems could provide further insights into the capabilities and limitations of both OSEM and BPL reconstruction methods. Moreover, studies should investigate the clinical impact of using BPL in various contexts, such as in the assessment of novel tracers or in different types of cancer. Understanding how BPL’s increased quantification translates into clinical benefits will be essential for its broader adoption.

## 5 Conclusion

While both OSEM and BPL reconstruction algorithms are effective for PET/CT imaging, BPL offers significant increases in several quantitative parameters, such as SUVmax and TBR. Strong correlations were observed between the two reconstruction methods; however, the wide limits of agreement observed for certain metrics, particularly TBR in PSMA imaging, suggest that these methods may not be directly interchangeable in longitudinal studies. Harmonization strategies, such as reconstruction-specific scaling factors or reference ranges, may help mitigate inter-method variability and improve consistency across imaging protocols. For longitudinal studies, consistent use of the same reconstruction method is recommended to ensure reliable quantification. Further research should optimize BPL implementation, including tracer-specific harmonization, and validate its impact on clinical decision-making across diverse populations and scanner platforms.

## Data Availability

The raw data supporting the conclusions of this article will be made available by the authors, without undue reservation.
